# Supplementary material for: Quantitative analysis of self-organized patterns in ombrotrophic peatlands
Source: Sci Rep. 2019 Feb 6;9:1499. doi: 10.1038/s41598-018-37736-8 (PMC6365544; doi:10.1038/s41598-018-37736-8)
Supplement: Supplementary file 1 — Supplementary Information [file 41598_2018_37736_MOESM1_ESM.pdf]

Quantitative analysis of self-organized patterns in ombrotrophic peatlands – Supplementary information

Chloé Béguin[1,2], Maura Brunetti[1,2], and Jérôme Kasparian[1,2,*]

[1]Group of Applied Physics, University of Geneva, Chemin de Pinchat 22, 1211 Geneva 4, Switzerland
[2]Institute for Environmental Sciences, University of Geneva, bd Carl Vogt 66, 1211 Geneva 4, Switzerland
[*]jerome.kasparian@unige.ch

ABSTRACT

This Supplementary Material provides captions for Supplementary Movies 1 and 2

Supplementary Movies captions

Supplementary Movie 1: Temporal evolution of vascular and *Sphagnum* mosses biomass, nutrient availability and hydraulic head over 400 years in an ombrotrophic peatland. Initial conditions and parameters as specified in the Methods section and in Table 1. Nutrient input N_{in} =2.5 g/m^2/yr. Each panel represents a square 256-meters wide area, with 2 m resolution.

Supplementary Movie 2: Temporal evolution of vascular plant patterns in an ombrotrophic peatland area of 256 by 256 m, after the switch to the 2$\times$ CO_2 scenario of GCMII, for the values of N_{in} corresponding to the different regions of Figure 3: N_{in} = 0.25, 1.25, 2.25, 3, 3.875, and 4.5 g/m^2/yr. Years 415–424 correspond to a drought, with a drop in precipitation to 50 mm/yr.
